# Exploring the influence of anionic lipids in the host cell membrane on viral fusion

**DOI:** 10.1042/BST20240833

**Published:** 2024-12-19

**Authors:** Daniel Birtles, Jinwoo Lee

**Affiliations:** Department of Chemistry and Biochemistry, University of Maryland, College Park, MD 20742, U.S.A.

**Keywords:** BMP, glycoproteins, lipids, membrane fusion, virology

## Abstract

Membrane fusion is an essential component of the viral lifecycle that allows the delivery of the genetic information of the virus into the host cell. Specialized viral glycoproteins exist on the surface of mature virions where they facilitate fusion through significant conformational changes, ultimately bringing opposing membranes into proximity until they eventually coalesce. This process can be positively influenced by a number of specific cellular factors such as pH, enzymatic cleavage, divalent ions, and the composition of the host cell membrane. In this review, we have summarized how anionic lipids have come to be involved in viral fusion and how the endosomal resident anionic lipid BMP has become increasingly implicated as an important cofactor for those viruses that fuse via the endocytic pathway.

## Introduction

Viruses are obligate intracellular parasites that require the biological machinery of the host/target cell in order to fulfill their lifecycle. The viral lifecycle can be simplified into four stages, the first of which is viral entry, which identifies the host cell and delivers the viral genome [1,2]. Once the virus’ genetic information has entered the host cell, replication can occur to produce nascent viral genomes and proteins, which are subsequently packaged together into the mature virion in a process known as maturation [3]. Finally, the release of the virion allows the infection to spread, ultimately leading to a disease state within the host organism [4]. Viruses have become incredibly well adapted to taking advantage of what is provided to them within the host cell, in essence turning the hosts own biological make up against it. The ability of viruses to hijack the replication machinery and utilize host cell receptors is well established in the literature [5–7], yet there is a growing body of work regarding how the host cells lipid composition impacts viral fusion, some of which will be discussed within this review. Gaining a more complete understanding of how viruses interact with and manipulate the constituents of our own cells is essential for both drug discovery and pandemic preparedness [8].

Viral entry encompasses two fundamental processes, receptor binding and membrane fusion, which occur in a stepwise manner to identify the host cell and facilitate the delivery of the viral genome, respectively [2,9]. How successful a virus may be in accomplishing either of these events can be dependent on several specific molecular factors, such as the presence of cellular receptors, localization of specific enzymes, the pH environment, divalent ions or the lipid composition of the host cell membrane [10–13]. Hence, it is the understanding of how and why a virus may require these molecular events that is integral for the development of novel therapeutics against emerging, lethal viruses. This review will primarily focus on how anionic lipids have come to be implicated as cofactors within the process of viral membrane fusion across a range of different viruses. Specifically, we will highlight the role of the viral fusion domains (FDs), a short, relatively hydrophobic sequence within the viral glycoprotein that interacts with the target membrane, and how it can be modulated by specific lipids, particularly the endosomal resident anionic lipid bis(monooleoylglycero)phosphate (BMP), to impact the success of viral fusion to great effect.

## Overview of viral fusion

Enveloped viruses require the merging of the viral and host cell membranes to deliver the viral genome into the host cell, which can occur at either the plasma or endosomal membrane [14]. Following initial contact between two opposing membranes, a hemi-fusion intermediate is formed where the outer monolayers begin to merge with one another whilst the inner layers remain intact, preventing any exchange of content [15–17]. This transitional stage, which contains a substantial energetic barrier, must be overcome to create the fusion pore where both leaflets of the membrane coalesce to allow the inner content of the virus (i.e. the viral genome) to mix with that of the host cell.

**Figure 1.**
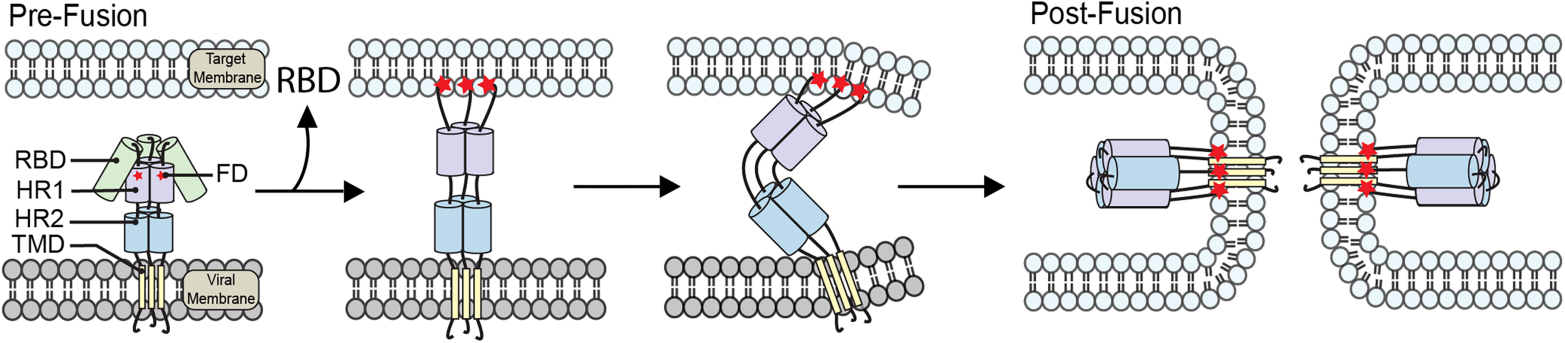
General mechanism of viral fusion. The viral glycoprotein contains a receptor binding domain (RBD), heptad repeats 1 (HR1), HR2, a fusion domain (FD) and is embedded in the mature virion via its transmembrane domain (TMD). Following a triggering event, the RBD then dissociates, allowing a conformational change to take place releasing the FD. The viral FD then embeds within the target cells membrane, forming a molecular bridge that physically connects the two opposing membranes. HR1 and HR2 domains then undergo complimentary interactions with one another, pulling the opposing membranes into proximity until they eventually coalesce. This creates a productive fusion pore, a toroidal corridor that allows the viral genome to pass from the virus into the target cell.

To facilitate fusion, viruses have developed highly specialized and efficient molecular machinery in the form of viral glycoproteins which participate in specific interactions and undergo large scale conformational changes [18,19]. These proteins can be categorized into one of three structural classes (I, II, and III), with class I expressed on the surface of the mature virion as a predominately alpha helical homo-trimer [20]. All known Class II glycoproteins exist as relatively short homo-dimers with high β-sheet content in their pre-fusion conformation, that then transition to a homo-trimer following engagement with the host cells membrane [21]. Alternatively, class III glycoproteins contain a mixture of the two secondary structures and are expressed as homo- or hetero-dimers on the viral surface [22]. Each monomer consists of multiple domains, which can vary in size and complexity, however regardless of class each generally contains a receptor binding domain (RBD), heptad repeat 1 (HR1), heptad repeat 2 (HR2), a FD and a transmembrane domain (TMD). Fusion is triggered by a variety of specific environmental factors which are largely conserved throughout the viral family. Triggers such as low pH (Influenza [10,23]), receptor binding (HIV [24]) and enzymatic cleavage (SARS-CoV-2 [13,25,26]), initiate a significant conformational change within the viral glycoprotein, which releases the FD (Figure 1). Once unsheathed, the FD subsequently buries within the host membrane perturbing the local lipid environment and producing the pre-hairpin intermediate [27,28]. This intermediate serves as a molecular bridge between the viral membrane and the host cell membrane, via the glycoproteins TMD and FD respectively [29]. Once tethered to both membranes the bridge then collapses in on itself, driven by complimentary interactions between the helical HR1 and HR2 domains. This action results in the formation of a trimer of hairpins, and in turn, the merging of opposing membranes [30]. This final state is thought to be the most energetically stable conformation of the viral glycoprotein, aiding the virus in overcoming the high energetic barrier associated with membrane fusion [31,32]. Although the fundamental principles behind viral fusion have been known for decades, it is only in recent years due to the advances in cryo-electron microscopy that these structures have been able to be visualized [33–36]. The ability to capture and solve intermediate structures to atomic resolution has provided a new level of detail to our understanding of the molecular mechanisms that govern viral fusion.

When fusion is triggered, the primary objective for the virus in that moment is to release the FD, a moderately hydrophobic sequence that is responsible for embedding itself within and perturbing the host cells lipid membrane [37–39]. All known viral glycoproteins contain such a domain which is often buried/hidden within the protein prior to fusion, and this safeguarding of the FD, along with high levels of sequence conservation reiterate its importance to the viral lifecycle. Once again, distinguishing structural features arise based on the previously introduced classification system. Class I FDs contain high amounts of conformational flexibility and exist at or near the N-terminus of the fusion subunit in the form of an N-terminal fusion peptide (FP) like that witnessed in influenza or HIV [40,41], an internal fusion loop (FL) as found in Ebola [42] or a combination of the two as seen in SARS-CoV-2 and Lassa virus (LASV) [43,44]. Class II contains only FLs such as those seen within rubella and Dengue virus’, however, these loops are significantly more rigid than those found in class I and generally operate through the formation of a hydrophobic patch [12,45]. Glycoproteins such as the class III G protein in vesicular stomatitis virus (VSV) contain two small FLs at the tips of beta sheets, requiring key aromatic amino acids to create a hydrophobic surface for membrane association in a bipartite manner [22,46,47]. Despite their differences, each class of viral FDs can be thought of as the first point of contact between the viral glycoprotein and the membrane lipids of the host cell. Thus, it comes as no surprise that the membrane lipid composition of the host cell has been shown to significantly impact the structure and/or function of numerous viral FDs [11,48–51].

## Role of anionic lipids

**Figure 2.**
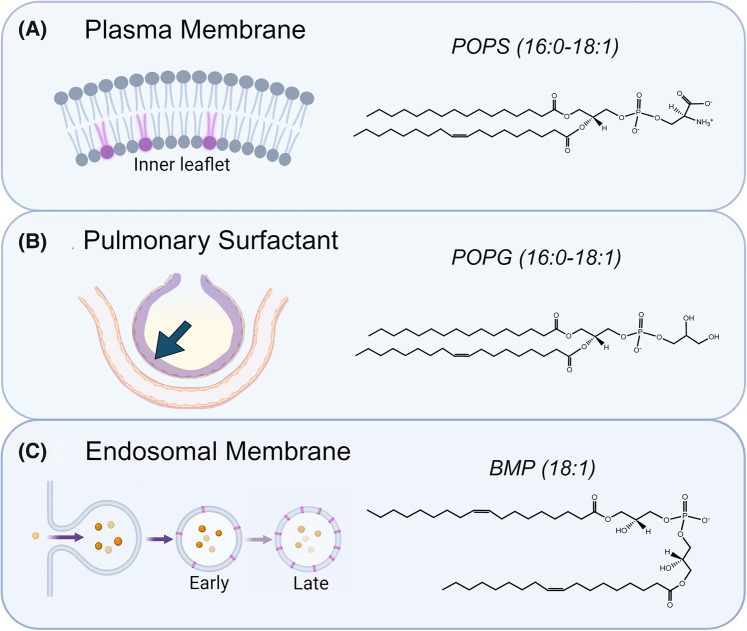
Localization of anionic lipids. (**A**) Phosphatidylserine (PS) is found within the inner leaflet of the plasma membrane (∼10%). (**B**) The epithelial cells within alveoli are lined with pulmonary surfactant to reduce surface tension and this liquid mixture contains phosphatidylglycerol (PG) (∼10%). (**C**) During the process of endocytosis, the concentration of the anionic lipid bis(monooleoylglycero)phosphate (BMP) in the endosomal membrane increases, reaching levels up to ∼20% in the late endosome/early lysosome. It should be noted that various isoforms of phosphoinositide's (PI) also exist across all three of the highlighted locations, although at lower concentrations than those highlighted above. Created in BioRender.

Viral fusion can be considered a dynamic process driven by selective pressure imparted by the target cells environment. Such pressures have driven viral evolution to require various cofactors that ensure the correct timing and location of fusion events, preventing the premature activation of viral glycoproteins. Anionic lipids are an example of a viral cofactor that has been found previously to be essential to the viral lifecycle of several deadly viruses. In mammalian cells, anionic lipids commonly reside in the inner leaflet of the plasma membrane in the form of phosphatidylserine (PS; ∼10%) and various different isomers of phosphoinositide's (PI; ∼2%) (Figure 2A) [52,53]. Such lipids are routinely found, facing the inside of the cell where they play key roles in intracellular signaling pathways, secretory systems, and the regulation of membrane protein function [54–56]. Due to the localization of anionic lipids to the inner leaflet of the plasma membrane, there is little likelihood that they could directly impact fusion through interacting with the viral glycoprotein. However, an indirect effect could still be possible through the ability of the anionic lipids to modulate the mechanical properties of the membrane (i.e. curvature), local pH or receptor localization. The reason for this distinction between direct and indirect effects is based upon the fact that the majority of viral FDs only interact with the outer leaflet of the membrane during the fusion process. An example of this can be seen with two of the most well studied viral FDs, the FP of GP41 from HIV and the FP of hemagglutinin (HA) from influenza, both of which are thought to only embed within the outer leaflet of the lipid bilayer [48,57]. This mechanism of shallow insertion still provides significant enough perturbation of the local membrane environment to initiate fusion, and several other viral FDs have been found to operate in a similar manner [45,58–62]. As a result of this mechanism, it comes as no surprise that there is little evidence of direct interactions between PS or PI on the inner leaflet of the plasma membrane and a viral FD during fusion.

Phosphatidylglycerol (PG) is another anionic lipid found within mammals, most commonly utilized within cells as a precursor for the synthesis of cardiolipin in the mitochondria (CL) and BMP in the endosomal pathway [63–65]. Alongside PI, PG also exists within pulmonary surfactant (Figure 2B), a liquid mixture predominately made up of phospholipids (∼90%) as well as proteins (∼10%) which coats the alveoli to reduce surface tension within the lungs [66]. With high localized concentrations (PG: ∼10 mol%, PI: ∼3 mol%), a potential role for such anionic lipids within the entry process of respiratory viruses is plausible [67]. However, pulmonary surfactant containing PG and PI has been shown to display anti-viral properties against different strains of influenza, SARS-CoV-2, and respiratory syncytial virus (RSV) [68–72]. With Influenza and RSV, the anionic lipids were found to directly interact with the mature virions and disrupt their initial interaction with the host cell [68]. Such interactions between the anionic lipid within the surfactant and viral glycoproteins may serve to trigger the conformational change necessary for fusion prematurely without the membrane in close enough proximity for the action to be productive. Alternatively, the antiviral properties of surfactant exhibited against SARS-CoV-2 were deemed to not be due to prevention of virus-host interactions, but rather some other, currently unknown molecular mechanism [68]. The anionic lipid components of pulmonary surfactant appear to be a promising area of research for therapeutic development, yet requires further research to unearth the molecular detail behind these antiviral properties.

Viruses can fuse with host cells at either plasma or endosomal membranes, and whilst the anionic lipid components of the plasma membrane may be redundant within viral fusion, that is not the case for the endosomal membrane. BMP (also known as lysobisphosphatidic acid (LBPA)) is an anionic lipid that is almost exclusively found in the membranes of endosomal and lysosomal vesicles (Figure 2C) [73–75]. A regulatory lipid involved in cholesterol transport, sphingolipid degradation, membrane remodeling and several other elements of lysosomal function, concentrations of up to 20 mol% can be found in the latter stages of the endocytic pathway [74–76]. It should be noted that several different isoforms of PI also exist within the endocytic pathway where they play important roles across a range of diverse biological processes, contributing towards the net negative charge of the membrane. However, any specific contribution towards viral fusion by PI isoforms are currently not clear [77]. Alongside its negative charge, BMP also contains a very unique sn-1;sn-1′ headgroup configuration that is not found in any other mammalian glycerophospholipid [78]. This headgroup stereochemistry, as opposed to the more traditional sn-3:sn-1′ orientation, is thought to confer stability in the extremely hydrolytic environment of the lysosome, and thus can be seen as a key structural feature [79]. Along with a novel headgroup structure, the most common isoform of BMP within mammalian cells also contains two unsaturated oleic acid tails (18:1), giving the lipid a cone shape which induces negative spontaneous curvature [73]. Together, these structural features alongside the negative charge of BMP create a distinctive lipid profile that can influence the biophysical properties of the membrane.

## BMP and viral fusion

Viruses such as influenza, LASV, Dengue, VSV and SARS-CoV-2, rely on specific molecular triggers found exclusively within the endosomal pathway to initiate fusion. Over the past decade BMP has become increasingly implicated as a co-factor within this process due to its unique structure, high relative abundance, and trans-bilayer distribution [80–84]. Whilst much progress has been made in understanding how viruses are able to take advantage of the unique traits of BMP, several gaps in our knowledge still remain.

BMP was first discovered as a potential contributor towards viral fusion within Dengue virus, and this was almost immediately followed by the finding of its influence on VSV [49,83]. For Dengue virus, both viral attachment and fusion is mediated by the envelope (E) protein, a class II viral glycoprotein that fuses at the late endosomal membrane [45]. Fusion is triggered via low pH, which brings about a conformational change in the E protein, that may only lead to a successful fusion event in the presence of anionic lipid [49]. The interaction between E protein and BMP was found to be via the FL, although molecular dynamic simulations revealed two other, well conserved sites of interaction within the fusion mechanism, however they were not specific to BMP. Furthermore, alongside Dengue other members of the flavivirus family such as Zika, Japanese encephalitis, and tick-borne encephalitis are predicted to share a similar mechanism with a requirement for specific interactions between BMP and the FP necessary for efficient fusion [85]. The VSV G protein is a class III viral glycoprotein, which undergoes a conformational change to initiate fusion when the pH is lowered, however this change is reversible at neutral pH in the absence of a membrane [46,61]. Moreover, the G protein is triggered to initiate fusion in the early endosome, but successful fusion events do not occur until the late endosome/early lysosome where significant amounts of BMP are known to accumulate [83,86]. This mechanism suggests that although the G protein undergoes a structural transition at low pH, its FL's may require the presence of BMP to interact with the membrane. Therefore, there is evidence that class II and class III viral glycoproteins in specific instances may require interactions with BMP to initiate membrane fusion.

The most common class of viral fusion machinery is that of class I, within which a trend has recently emerged amongst members that utilize the endocytic pathway such as SARS-CoV-2, influenza, and LASV, whereby BMP serves as a co-factor to promote membrane fusion. Hemagglutinin, the glycoprotein of influenza, was first discovered to be positively impacted by anionic lipids almost forty years ago [87,88]. Then in 1997 Chernomordik et al. [89] discovered that the shape of the lipids in question also had an effect, with the cone-shaped oleic acid found to promote membrane fusion. BMP contains both a negative charge and a cone shape, yet it took another 25 years to establish that BMP modulated the local lipid environment to positively influence influenza fusion [81]. Unlike influenza which can only enter cells in the low pH environment of the endocytic pathway, SARS-CoV-2 can enter cells through both plasma and endosomal membranes, however a preference for the latter exists due to both decreased pH and increased anionic lipid concentration [90,91]. Research focusing on the SARS-CoV-2 FD revealed that BMP promoted fusion more readily than PG or PS, not due to specific interactions from its unique headgroup but rather its ability to impact membrane fluidity [80]. BMP was found to increase membrane fluidity in large unilamellar vesicles when compared to POPS, reasoned to be due to a decrease in hydrogen bonding between the headgroup and PC lipids, as well as the increased unsaturation in its aliphatic chains (BMP: 18:1, PS: 16:0–18:1) [80,92]. Whilst evidence for the involvement of BMP in influenza and SARS-CoV-2 fusion was acquired *in vitro*, LASV was discovered to utilize BMP for successful fusion events *in vivo* [82]. Markosyan et al. found that BMP plays a critical role in facilitating the transition from hemi-fusion to full fusion, which serves as a significant energetic barrier to productive fusion. Moreover, the anionic lipid also served to aid the growth of fusion pores. Due to the nature of the study, the researchers could only hypothesize about the means by which this takes place, suggesting either specific interactions between the lipid and glycoprotein aiding in structural rearrangements of the fusion machinery, or destabilization of the hemi fusion intermediate [82].

To summarize, BMP appears to assist the viral fusion process through two mechanisms. (1) BMP can directly interact with the viral FD and/or other regions of the viral glycoprotein to aid with the conformational changes necessary for successful fusion events. (2) Increasing concentrations of BMP can alter the mechanical properties of the membrane, possibly indirectly promoting the transition from hemi-fusion to full fusion. It remains unclear whether these mechanisms may only operate independently, or whether they can simultaneously aid viral fusion. Further research is required to understand the molecular intricacies at play.

## Outlook

Over the past 50+ years significant research has been conducted in order to improve our understanding of how viral glycoproteins operate on a molecular level to initiate membrane fusion [11,18,24,28,31,61,93–98]. Unfortunately, significantly less attention has been given to the integral and specific role of the membranes within this process. Particularly, how the lipid constituents and physical properties of such membranes may aid the viral glycoproteins in eliciting fusion and thus, large gaps remain in our understanding. BMP is a unique anionic lipid found within the endocytic pathway that can alter the mechanical properties of membranes to positively regulate viral fusion. With the number of viruses being associated with BMP only increasing over recent years, it stands to reason that there could yet be many more. The singular localization of BMP to the endocytic pathway alongside its unique headgroup stereochemistry makes the anionic lipid a promising drug target. These features in particular could serve to alleviate unintended, off-target effects as well as aid the affinity and specificity of designer small molecule inhibitors. A lipid based therapeutic preventing the interactions between the virus and BMP, or using BMP as a biomarker itself for drug delivery may be possible.

To move forward with therapeutic development, structural information in physiologically relevant membranes that can represent the lipid composition, asymmetry, and fluidity of an endosomal membrane is integral. This would go some way to understanding not only the specific atomic interactions at play between proteins and lipids, but also the location within the molecular mechanism that anionic lipids positively impact fusion. While advances in both cryo-EM and NMR have enabled us to visualize membrane proteins with unprecedented detail, most of these proteins continue to pose significant experimental challenges. Thus, a combined experimental and computational approach would be well-suited to this complex problem, not only for structural elucidation but also with regards to drug design. *De novo* design of high affinity binders through the use of artificial intelligence is a burgeoning field that will more than likely significantly contribute to a wide array of scientific fields over the preceding years [99–103]. However, it remains integral that alongside structural information and drug design, their also exists model systems for the study of viral fusion on a biophysical level [104–106]. Such systems can offer controlled, simplified environments that allow researchers to isolate and study specific aspects of the fusion process in great detail, often providing the means by which to screen drug candidates in a high throughput manner that would be impossible *in vivo*.

## Perspectives

- Membrane fusion is an integral component of the lifecycle for enveloped viruses, a process which relies on highly conserved glycoproteins found on the exterior of the mature virion. Such proteins can require specific environmental factors to initiate fusion such as pH, enzymatic cleavage, and anionic lipids.
- The anionic lipid BMP, found within the late endosome has been implicated with increasing incidence as a key factor contributing towards the initiation of viral fusion in several different viral families.
- Structural information of viral fusion machinery in physiological relevant membranes would greatly enhance our current understanding of how these viruses initiate and facilitate membrane fusion *in vivo.* Understanding the role played by anionic lipids, specifically BMP, within that mechanism in molecular detail is key for therapeutic development.

## Abbreviations

BMP: bis(monooleoylglycero)phosphate
FD: fusion domain
FL: fusion loop
FP: fusion peptide
HR1: heptad repeat 1
HR2: heptad repeat 2
LASV: Lassa virus
PG: phosphatidylglycerol
PI: phosphoinositide
PS: phosphatidylserine
RBD: receptor binding domain
RSV: respiratory syncytial virus
TMD: transmembrane domain
VSV: vesicular stomatitis virus
