# Correction: Exploring the influence of anionic lipids in the host cell membrane on viral fusion

**DOI:** 10.1042/BST20240833_COR

**Published:** 2025-04-28

**Keywords:** BMP, glycoproteins, lipids, membrane fusion, virology

The authors would like to correct their published article to reflect that their study was supported by funding from the National Science Foundation (NSF), grant number CHE-2238139.

